# State-Level Variation in Waitlist Mortality and Transplant Outcomes Among Patients Listed for Heart Transplantation in the US From 2011 to 2016

**DOI:** 10.1001/jamanetworkopen.2020.28459

**Published:** 2020-12-09

**Authors:** Emmanuel Akintoye, Doosup Shin, Paulino Alvarez, Alexandros Briasoulis

**Affiliations:** 1 Division of Cardiovascular Diseases, University of Iowa Hospitals and Clinics, Iowa City; 2 Division of Heart Failure and Cardiac Transplantation Section, Cleveland Clinic, Cleveland, Ohio

## Abstract

**Importance:**

Little is known about geographic variation in the outcomes of adult patients listed for heart transplantation in the US. Identifying the patterns and extent of variation is important to minimize disparity in outcomes.

**Objective:**

To evaluate the geographic patterns, extent, and factors associated with state-level variation in outcomes of adult patients listed for heart transplantation in the US.

**Design, Setting, and Participants:**

This nationwide retrospective cohort study used data from the United Network for Organ Sharing database to identify adult patients listed for heart transplantation at status 1A between January 1, 2011, and December 31, 2016. Patients were followed up until March 31, 2018. Data were analyzed from November 1, 2019, to September 19, 2020.

**Main Outcomes and Measures:**

The study evaluated state-level variation in the 3 main organ transplant measures: waitlist mortality, transplant rate, and risk-adjusted 1-year graft survival. The rate of death while on the waitlist and the rate of transplant were calculated for each state per 1000 waitlist person-days listed at status 1A over the study period. Risk-adjusted 1-year graft survival was calculated based on the Scientific Registry of Transplant Recipients risk-adjustment model. State-level variation in each outcome measure was evaluated via multivariable-adjusted models.

**Results:**

Across 50 states and the District of Columbia, a total of 15 036 patients (mean [SD] age, 52 [13] years; 3531 women [24%]; 9626 White [64%]) were listed at status 1A for adult heart transplantation between 2011 and 2016. Of those, 2146 patients (14.3%) died while on the waitlist, and 10 982 patients (73.0%) received transplants. Among those who received transplants, the median time on the waitlist was 31 days (interquartile range, 13-61 days). State-level outcomes ranged from 1.0 to 7.8 deaths per 1000 waitlist person-days for waitlist mortality, 5.6 to 34.5 transplants per 1000 waitlist person-days for transplant rate, and 87% to 92% for risk-adjusted 1-year graft survival. In a comparison of the highest and lowest quartiles, significant state-level variation was found in waitlist mortality (hazard ratio [HR], 1.53; 95% CI, 1.27-1.86), transplant rate (HR, 1.57; 95% CI, 1.31-1.87), and 1-year graft survival (odds ratio, 2.07; 95% CI, 1.64-2.62).

**Conclusions and Relevance:**

The study’s findings indicate that significant state-level variation exists in the outcomes of patients listed for heart transplantation in the US. Identifying and addressing the factors associated with these geographic variations in outcomes is important to ensure a fair allocation system.

## Key Points

**Question:**

Do the outcomes of patients listed for heart transplantation in the US vary by geographic location?

**Findings:**

In this cohort study of 15 036 adult candidates for heart transplantation in the US, significant geographic variation was found for 3 main transplant measures (waitlist mortality, transplant rate, and 1-year graft survival) among patients listed for heart transplantation at status 1A between 2011 and 2016. Across the 50 states and the District of Columbia, waitlist mortality ranged from 1 to 8 deaths per 1000 waitlist person-days, transplant rates ranged from 6 to 35 transplants per 1000 waitlist person-days, and risk-adjusted 1-year graft survival ranged from 87% to 92%.

**Meaning:**

The study’s findings indicated significant state-level variation in the outcomes of patients listed for heart transplantation in the US.

## Teaser

This cohort study uses data from the United Network for Organ Sharing database to evaluate the geographic patterns, extent, and factors associated with state-level variation in outcomes of adult patients listed for heart transplantation in the US.

## Introduction

The diagnosis of heart failure implies a negative prognosis in the absence of optimal intervention. In the US, an estimated 6.2 million adults 20 years and older experienced heart failure between 2013 and 2016, and the prevalence of heart failure is expected to increase as the population ages over time.^1^ For patients with end-stage heart failure who received medical treatment, 1-year mortality was 75%.^2^ Furthermore, patients reliant on inotropic medications who received optimal medical therapy had a mortality rate of more than 50% at 1 year.^3^ Heart transplantation is the conclusive life-saving treatment for end-stage heart failure. Survival among patients with end-stage heart failure has been reported to substantially improve after transplantation, with a current median survival of 11 years.^4^

Although the number of candidates awaiting heart transplantation has substantially increased, transplant rates have not increased at the same pace. This situation has produced a worsening mismatch between supply and demand.^5^ Therefore, fair and efficient allocation of donor organs to candidates with the most medically severe conditions is important to improve outcomes. In the US, the allocation policy is regulated by the United Network for Organ Sharing (UNOS) and has been updated periodically to reflect changes in medical practice and to make up for shortcomings.^6^ However, ongoing concerns remain regarding geographic heterogeneity in transplant practices.^5,6,7^ For example, a previous analysis indicated considerable regional variation in waitlist time and the use of mechanical circulatory support as a transition to transplantation.^6^ There is currently limited evidence regarding the ways in which these variations in practice are associated with outcomes. To minimize potential interstate disparity in transplant outcomes, it is important to identify the patterns, extent, and factors associated with geographic variation. Therefore, this study aimed to investigate state-level variation in waitlist and posttransplantation outcomes using data from the UNOS database from 2011 to 2016.

## Methods

Data for this analysis were obtained from the UNOS database, which collects information on all organ donation and transplant events occurring within the Organ Procurement and Transplantation Network (OPTN), which is the organ transplant network in the US. The 50 states are divided into 11 OPTN regions, within which transplant-associated issues are coordinated. Extensive baseline information, including demographic characteristics, clinical and laboratory details at the time of waitlist entry, and prospective waitlist and posttransplant follow-up data were collected for each candidate. This study was approved by the institutional review board of The University of Iowa and considered exempt from informed consent because it was a secondary analysis of a deidentified data set. The study followed the Strengthening the Reporting of Observational Studies in Epidemiology (STROBE) reporting guideline for cohort studies.

For this analysis, we included data on adult patients 18 years and older who were listed at status 1A for heart transplantation between January 1, 2011, and December 31, 2016. Status 1A was defined as the receipt of temporary mechanical circulatory support (MCS) for acute hemodynamic support, MCS with objective evidence of substantial device-associated complications, continuous mechanical ventilation, or continuous infusion of high-dose or multiple intravenous inotropic medications with continuous hemodynamic monitoring.

The unit of comparison was the patient’s state of residency at the time of waitlist entry (for waitlist mortality) or the time of transplant (for transplant outcomes). For rare circumstances in which a patient moved out of the listing state during the study period, waitlist mortality was ascribed to the state of residency at the time of waitlist entry and transplant outcome was ascribed to the state of residency at the time of transplant. This information was available for each candidate in the UNOS database. Additional state-specific information, such as the number of transplant centers per state, was retrieved from the Scientific Registry of Transplant Recipients (SRTR), which contains additional information on transplant programs and organ procurement organizations. The SRTR is overseen by the Health Resources and Services Administration of the US Department of Health and Human Services. Information on population density for each state was derived from the US Census Bureau.

Patients on the waitlist were followed up from their date of entry into status 1A to their date of removal from the waitlist or the date on which their status was deescalated during the study period. Only waitlist time at status 1A was included in this analysis, and candidates who had been deescalated to a lower status were allowed to reenter the cohort if they were subsequently escalated to status 1A during the study period. For those who underwent heart transplantation, we included posttransplant follow-up data through March 31, 2018, to allow for a minimum of 1 year of follow-up for all patients.

We evaluated the 3 main measures of transplant outcomes: waitlist mortality, transplant rate, and risk-adjusted 1-year graft survival. To account for variable waitlist time among patients and to assess state-specific waitlist burden, time at risk was quantified using person-days at status 1A. For each state, the person-days represented the sum of waitlist time at status 1A (in days) for all patients during the study period. Mortality and transplant rates were expressed per waitlist person-days at status 1A. One-year graft survival was risk adjusted according to the SRTR national risk-adjustment model for posttransplant outcomes (eTable 1 in the Supplement).^8^ All end points were evaluated at the state level.

### Statistical Analysis

The total number of person-days on the waitlist at status 1A was calculated for each state. Thereafter, the states were divided into 4 quartiles based on their total waitlist person-days, and baseline patient characteristics were compared between the highest and lowest quartiles using a χ^2^ test for categorical variables and an analysis of variance or Kruskal-Wallis test (as appropriate) for continuous variables.

To evaluate interstate variation in waitlist mortality and transplant rates, we divided the states into quartiles based on the time to each end point. State-level variation in the time to each end point was then assessed by comparing the highest and lowest quartiles using multivariable-adjusted competing-risk regression analysis according to the subdistribution proportional hazards model of Fine and Gray.^9^ A transplant event was specified as a competing event in the analysis of waitlist mortality and waitlist mortality as a competing event in the analysis of transplant outcomes. We adjusted for important patient-specific characteristics, including age, sex, race and ethnicity, presence of diabetes, body mass index (calculated as weight in kilograms divided by height in meters squared), educational level, cigarette use, previous cancer, insurance status, markers of pretransplant illness severity (primary cardiac diagnosis and receipt of intravenous inotropic medications, temporary MCS, durable MCS, and mechanical ventilation at the time of waitlist entry), and state-specific characteristics (number of transplant centers per state and population density). Because transplant activities were coordinated within each OPTN region, SEs were clustered around each region to account for potential associations between observations within the region. The proportional hazards assumption was tested and found to be valid across the state quartiles for both the mortality and transplant analyses.

State-level variation in 1-year graft survival among transplant recipients was similarly evaluated by dividing the states into quartiles based on their survival rates and comparing the highest and lowest quartiles using a multivariable-adjusted mixed-effects logistics model. Covariates that were part of the national SRTR risk-adjustment model were included as fixed effects in the model, and state OPTN region was specified as a random effect in the model (eTable 1 in the Supplement).

We evaluated independent factors associated with the 3 end points using similar variables and models (ie, a proportional hazards model was used for waitlist mortality and transplant rate, and a mixed-effects logistics model was used for 1-year graft survival). For the proportional hazards models, the assumption of proportionality was valid for all variables, with only a few exceptions (for the waitlist mortality model, the receipt of durable MCS; for the transplant model, sex, body mass index, the receipt of inotropic medications at the time of waitlist entry, and population density). Nonproportionality among these variables was therefore addressed by including an interaction term with time for each variable. Data were complete for all variables, with the exception of primary cardiac diagnosis at the time of waitlist entry (299 patients [2.0%]) and educational level (901 patients [6.0%]) for the waitlist mortality and transplant analyses, and pulmonary artery systolic pressure (324 patients [3.0%]) and capillary wedge pressure (986 patients [9.0%]) for the 1-year survival analysis. For each of these variables, we performed multiple imputations using the data augmentation algorithm of the Markov Chain Monte Carlo procedure, which imputes missing values by drawing from a conditional distribution of the missing values based on the observed data. Ten imputations were performed for each missing value. We performed a sensitivity analysis based on a complete-case analysis to assess the robustness of our estimates.

All analyses were performed using Stata software, version 16 (StataCorp), with a 2-tailed significance threshold of *P* < .05. Data were analyzed from November 1, 2019, to September 19, 2020.

## Results

**Figure 1.**
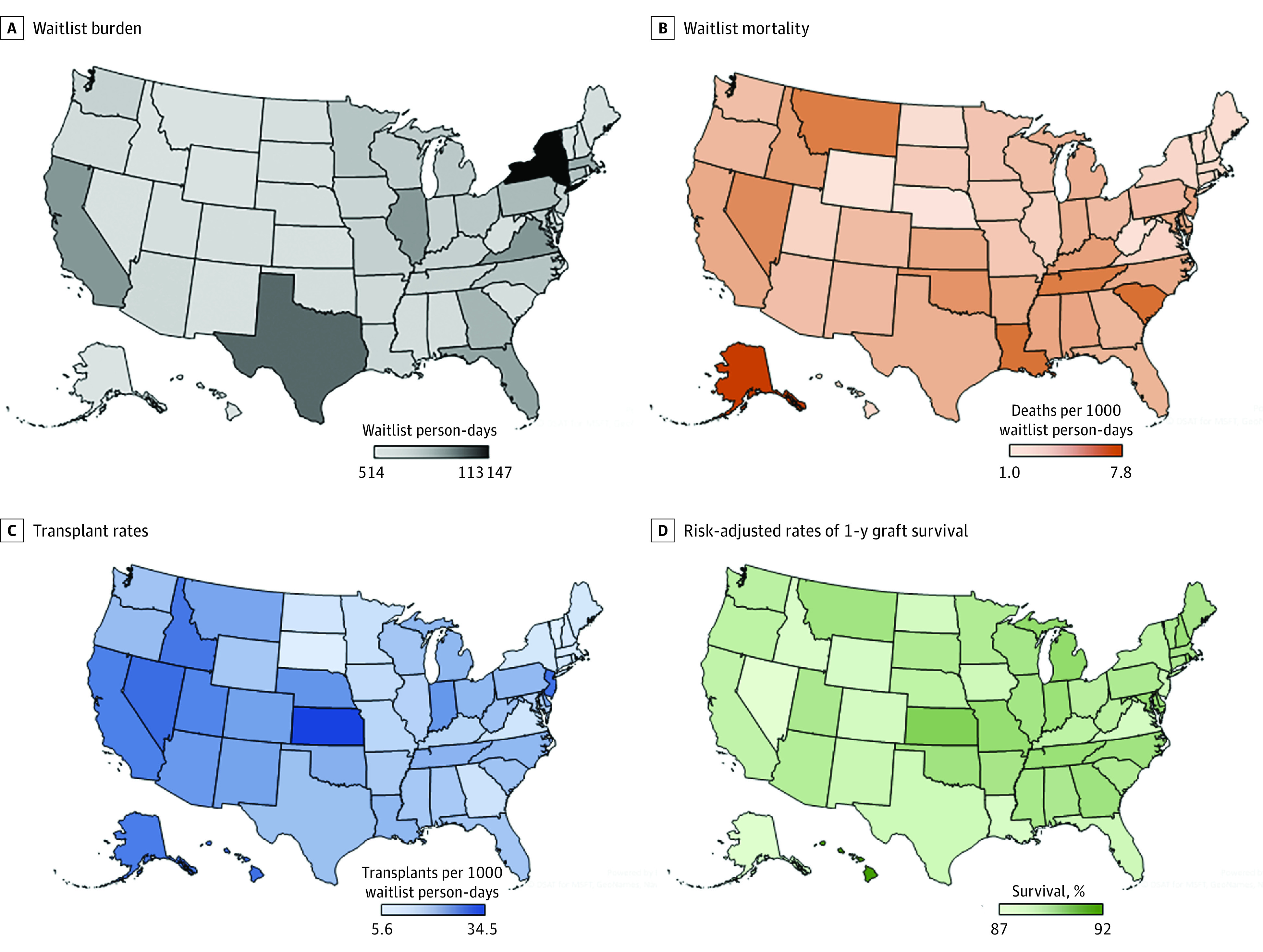
State-Level Outcomes A, Variation in waitlist person-days among patients listed at status 1A for heart transplant in the US. The waitlist burden ranged from 514 waitlist person-days in Alaska to 113 147 waitlist person-days in New York (national median, 7489 waitlist person-days; IQR, 3844-19 105 waitlist person-days). B, Waitlist mortality per 1000 waitlist person-days at status 1A. Estimates ranged from 1 death per 1000 waitlist person-days in Nebraska and Wyoming to 7.8 deaths per 1000 waitlist person-days in Alaska (national median, 2.9 deaths per 1000 waitlist person-days; IQR, 1.8-3.7 deaths per 1000 waitlist person-days). C, Transplant rates per 1000 waitlist person-days at status 1A. Estimates ranged from 5.6 transplants per 1000 waitlist person-days in Vermont to 34.5 transplants per 1000 waitlist person-days in Kansas (national median, 15.7 transplants per 1000 waitlist person-days; IQR, 9.2-21.3 transplants per 1000 waitlist person-days). D, Risk-adjusted rates of 1-year graft survival. Survival estimates ranged from 87% in Nevada, Arkansas, Idaho, Wyoming, and Louisiana to 92% in Hawaii (national median, 89%; IQR, 88%-89%).

Across 50 states and the District of Columbia, a total of 15 036 candidates (mean [SD] age, 52 [13] years; 3531 women [24%]; 9626 White [64%]) were listed at status 1A for adult heart transplantation between 2011 and 2016 (Table 1). State-level variation in waitlist burden across the country is shown in Figure 1A. Notably, one-third of the national person-days on the waitlist were from 4 states: New York, Texas, Virginia, and California. An analysis of the highest and lowest quartiles of state-level person-days on the waitlist indicated that patients in the highest quartile were more likely to be greater than or equal to 65 years (18.3% vs 11.9%, respectively), to be receiving inotropic medications at the time of waitlist entry (34.6% vs 29.6%), and to have Black (26.5% vs 19.5%) or Hispanic (12.6% vs 4.3%) ancestry, diabetes (33.1% vs 28.0%), and Medicare insurance coverage (32.6% vs 30.3%) compared with patients in the lowest quartile. Patients in the highest quartile were less likely to have White ancestry (56.1% vs 72.7%) and private insurance coverage (49.7% vs 53.4%) than those in the lowest quartile.

**Table 1.** Baseline Participant Characteristics

Characteristic	No. (%)					*P* value^a^
	State-level quartile for person-days on waitlist				Total (N = 15 036)	
	Lowest (n = 3928)	2nd (n = 3861)	3rd (n = 3954)	Highest (n = 3293)		
Person-days on waitlist at status 1A during study period, mean (SD)^b^	8171 (4259)	20 550 (3986)	42 749 (5415)	81 035 (25 949)	36 400 (29 726)	<.001
Age, y						
Mean (SD)	51 (13)	52 (12)	52 (13)	53 (13)	52 (13)	<.001
Category						
<65	3459 (88.1)	3290 (85.2)	3276 (82.9)	2691 (81.7)	12716 (84.6)	<.001
≥65	469 (11.9)	571 (14.8)	678 (17.2)	602 (18.3)	2320 (15.4)	
Female sex	949 (24.2)	930 (24.1)	900 (22.8)	752 (22.8)	3531 (23.5)	.29
Race/ethnicity						
White	2856 (72.7)	2635 (68.3)	2287 (57.8)	1848 (56.1)	9626 (64.0)	<.001
Black	767 (19.5)	979 (25.4)	933 (23.6)	873 (26.5)	3552 (23.6)	
Hispanic	167 (4.3)	151 (3.9)	493 (12.5)	415 (12.6)	1226 (8.2)	
Asian	59 (1.5)	77 (2.0)	201 (5.1)	123 (3.7)	460 (3.1)	
Other	79 (2.0)	19 (0.5)	40 (1.0)	34 (1.0)	172 (1.1)	
Diabetes	1099 (28.0)	1203 (31.2)	1104 (27.9)	1091 (33.1)	4497 (29.9)	<.001
Cigarette use	1858 (47.4)	1912 (49.6)	1704 (43.2)	1543 (47.0)	7017 (46.8)	<.001
Creatinine, mean (SD), mg/dL	1.2 (0.6)	1.0 (0.4)	1.1 (0.4)	1.1 (0.4)	1.1 (0.5)	.33
BMI, mean (SD)	28 (5.5)	28 (5.0)	27 (4.9)	28 (5.1)	28 (5.2)	.007
Blood type group O	1815 (46.2)	1773 (46.2)	1824 (46.2)	1570 (47.7)	6982 (46.5)	.001
IV inotropic medication at waitlist entry	1164 (29.6)	1124 (29.1)	1517 (38.4)	1139 (34.6)	4944 (32.9)	<.001
Temporary MCS	357 (9.1)	412 (10.7)	346 (8.8)	329 (10.0)	1444 (9.6)	0.19
IABP	253 (6.4)	256 (6.6)	231 (5.8)	220 (6.7)	960 (6.4)	0.41
ECMO	70 (1.8)	97 (2.5)	83 (2.1)	75 (2.3)	325 (2.2)	0.15
Durable MCS^c^	1076 (27.4)	1218 (31.6)	845 (21.4)	929 (28.2)	4068 (27.1)	0.43
Mechanical ventilation	86 (2.2)	128 (3.3)	89 (2.3)	122 (3.7)	425 (2.8)	<0.001
College educational level	2061 (55.0)	2019 (55.2)	2149 (59.4)	1793 (57.8)	8022 (56.8)	0.02
Primary cardiac diagnosis						
Nonischemic cardiomyopathy	3579 (92.7)	3508 (92.5)	3496 (90.0)	2850 (88.4)	13433 (91.0)	NA
Ischemic cardiomyopathy	68 (1.8)	61 (1.6)	143 (3.7)	173 (5.4)	445 (3.0)	
Congenital heart disease	113 (2.9)	107 (2.8)	105 (2.7)	73 (2.3)	398 (2.7)	
Retransplant	100 (2.6)	115 (3.0)	139 (3.6)	128 (4.0)	482 (3.3)	
Insurance status						
Private	2100 (53.5)	2060 (53.4)	2040 (51.7)	1638 (49.7)	7842 (52.2)	<.001
Medicare	1189 (30.3)	1198 (31.0)	1208 (30.6)	1073 (32.6)	4668 (31.1)	
Medicaid	432 (11.0)	515 (13.3)	523 (13.2)	457 (13.9)	1927 (12.8)	
No. of transplant centers per state, mean (SD)	2 (1)	4 (2)	9 (4)	7 (2)	5 (4)	<.001
Population density per km^2^, median (IQR)	37 (21-61)	109 (68-236)	95 (90-141)	81 (39-161)	81 (39-110)	<.001

Abbreviations: BMI, body mass index (calculated as weight in kilograms divided by height in meters squared); ECMO, extracorporeal membrane oxygenation; IABP, intra-aortic balloon pump; IQR, interquartile range; IV, intravenous; MCS, mechanical circulatory support; NA, not available.

^a^ *P* values compare highest with lowest quartile.

^b^ To calculate the mean, the total number of person-days on the waitlist at status 1A was calculated for each state. Thereafter, the states were divided into 4 quartiles based on their total waitlist person-days. The mean number of person-days across states was then calculated within each quartile.

^c^ Durable MCS represents the use of a left or right ventricular assistive device or a total artificial heart.

**Figure 2.**
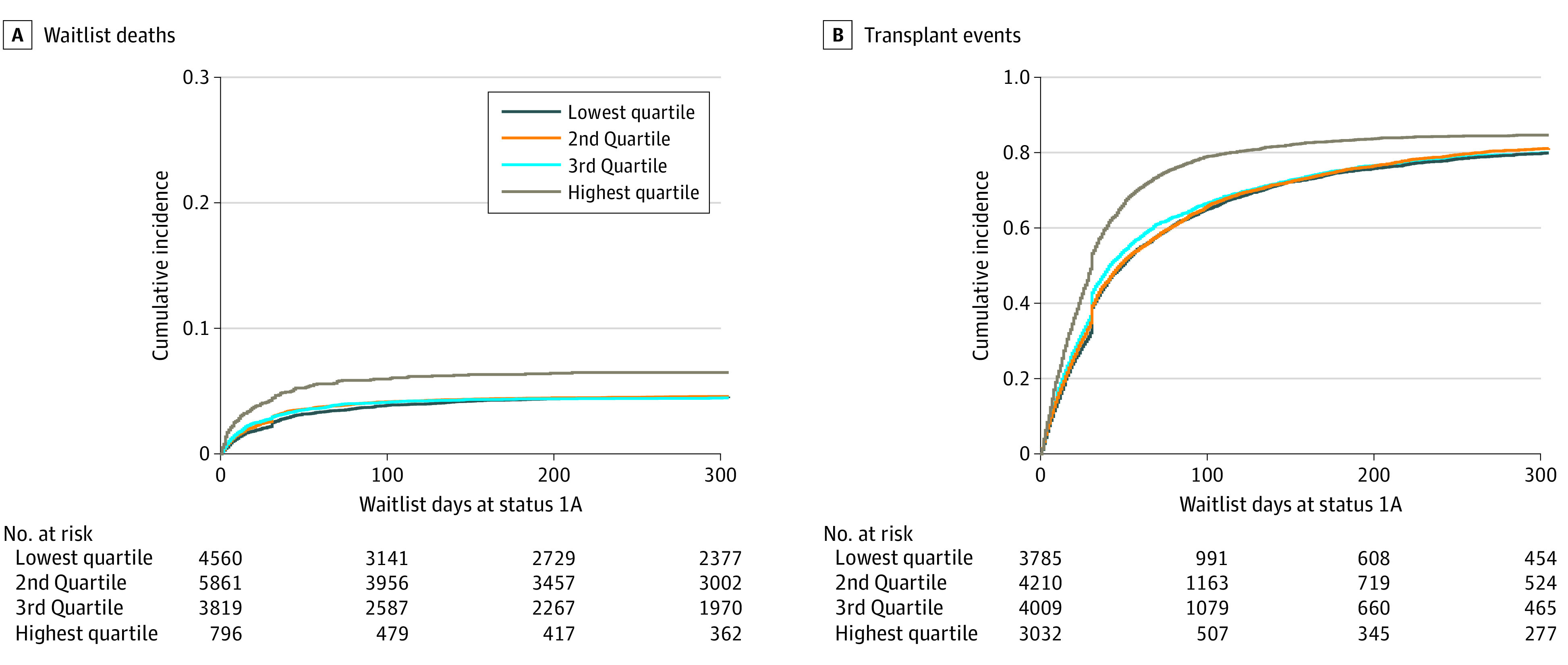
Cumulative Incidence of Waitlist Deaths and Transplant Events A, Cumulative incidence of waitlist deaths, stratified by state-level quartile (only the first 300 days of follow-up is shown). The median time from waitlist entry to death ranged from 13 days for the highest quartile to 30 days for the lowest quartile (national median, 20 days; IQR, 7-44 days). B, Cumulative incidence of transplant events, stratified by state-level quartile (only the first 300 days of follow-up is shown). The median time from waitlist entry to transplant ranged from 24 days for the highest quartile to 31 days for the lowest quartile (national median, 31 days; IQR, 13-61 days).

During the study period, 2146 patients (14.3%) died while on the waitlist, and 10 982 patients (73.0%) received transplants. The state of residency at the time of waitlist entry and the time of transplant was the same for most patients, with the exception of 255 individuals (1.7%) who had moved to a different state at the time of transplant. The rates of waitlist mortality ranged from 1.0 deaths per 1000 waitlist person-days in Nebraska and Wyoming to 7.8 deaths per 1000 waitlist person-days in Alaska (national median, 2.9 deaths per 1000 waitlist person-days; interquartile range [IQR], 1.8-3.7 deaths per 1000 waitlist person-days) (Figure 1B; eTable 2 in the Supplement). Significant variation in waitlist mortality rates was found when comparing the highest and lowest quartiles (hazard ratio [HR], 1.53; 95% CI, 1.27-1.86) (Figure 2A; eTable 3 in the Supplement). Among those who died while on the waitlist, the median time from waitlist entry to death ranged from 13 days (IQR, 4-36 days) for the highest quartile to 30 days (IQR, 9-64 days) for the lowest quartile (national median, 20 days; IQR, 7-44 days).

The transplant rate ranged from 5.6 transplants per 1000 waitlist person-days in Vermont to 34.5 transplants per 1000 waitlist person-days in Kansas (national median, 15.7 transplants per 1000 waitlist person-days; IQR, 9.2-21.3 transplants per 1000 waitlist person-days) (Figure 1C; eTable 2 in the Supplement). Significant state-level variation in transplant rates was found when comparing the highest and lowest quartiles (HR, 1.57; 95% CI, 1.31-1.87) (Figure 2B; eTable 3 in the Supplement). Among those who received a transplant, the median time from waitlist entry to transplant ranged from 24 days (IQR, 10-41 days) for the highest quartile to 31 days (IQR, 14-74 days) for the lowest quartile (national median, 31 days; IQR, 13-61 days). Between the 11 OPTN regions, the waitlist mortality rate ranged from 1.7 deaths to 3.8 deaths per 1000 waitlist person-days, and the transplant rates ranged from 7.2 transplants to 25.8 transplants per 1000 waitlist person-days. These regional differences explained 39% of the variability in the state-level mortality rates and 56% of the variability in the state-level transplant rates. Risk-adjusted 1-year graft survival ranged from 87% in 5 states (Nevada, Arkansas, Idaho, Wyoming, and Louisiana) to 92% in Hawaii (national median, 89%; IQR, 88%-89%), and there was significant state-level variation (odds ratio [OR], 2.07; 95% CI, 1.64-2.62) between the highest and lowest quartiles (Figure 1D; eTable 2 and eTable 4 in the Supplement).

When we evaluated other independent factors associated with outcomes, the presence of extracorporeal membrane oxygenation (ECMO) at the time of waitlist entry was the most significant factor associated with waitlist mortality (HR, 2.97; 95% CI, 2.25-3.92) and transplant rate (HR, 0.61; 95% CI, 0.48-0.77) (Table 2). Among the variables associated with both outcomes, the receipt of intravenous inotropic medications at the time of waitlist entry and the number of transplant centers per state had positive associations with mortality and transplant rates, while Black race, body mass index, congenital heart disease, the presence of ECMO, and the receipt of durable MCS, mechanical ventilation, and retransplant had negative associations. For example, Black race was associated with a higher rate of waitlist mortality (HR, 1.14; 95% CI, 1.02-1.27) but a lower rate of transplant (HR, 0.85; 95% CI, 0.81-0.89) compared with White race.

**Table 2.** Multivariable Factors Associated With Waitlist Mortality and Transplant

Variable	Waitlist mortality		Transplant	
	HR (95% CI)	*P* value	HR (95% CI)	*P* value
Age, y	1.01 (1.01-1.02)	.002	1.00 (0.99-1.01)	.25
Female sex	0.98 (0.87-1.09)	.87	1.08 (1.03-1.13)^a^	.001
Race/ethnicity				
White	1 [Reference]	NA	1 [Reference]	NA
Black	1.14 (1.02-1.27)	.02	0.85 (0.81-0.89)	<.001
Hispanic	0.96 (0.80-1.16)	.73	0.99 (0.93-1.07)	.81
Asian	0.78 (0.56-1.06)	.11	1.13 (1.01-1.27)	.04
Diabetes	1.07 (0.97-1.19)	.14	0.98 (0.94-1.03)	.52
BMI	1.02 (1.01-1.03)	<.001	0.97 (0.96-0.97)^a^	<.001
History of cigarette use	1.07 (0.87-1.18)	.14	0.94 (0.91-0.98)	.01
Previous cancer	0.96 (0.81-1.14)	.68	1.01 (0.93-1.08)	.84
IV inotropic medication at waitlist entry	1.11 (1.00-1.23)	.04	1.09 (1.04-1.16)^a^	.001
Temporary mechanical support at waitlist entry				
None	1 [Reference]	NA	1 [Reference]	NA
IABP	1.36 (1.14-1.63)	.001	0.98 (0.89-1.08)	.83
ECMO	2.97 (2.25-3.92)	<.001	0.61 (0.48-0.77)	<.001
Durable mechanical support at waitlist entry	0.46^b^ (0.40-0.54)	<.001	1.07 (1.02-1.11)	.006
Mechanical ventilation at waitlist entry	1.78 (1.36-2.34)	<.001	0.64 (0.54-0.77)	<.001
College educational level	0.95 (0.86-1.04)	.27	0.99 (0.95-1.03)	.84
Primary cardiac diagnosis				
Nonischemic cardiomyopathy	1 [Reference]	NA	1 [Reference]	NA
Ischemic cardiomyopathy	1.01 (0.76-1.32)	.96	0.96 (0.86-1.07)	.51
Congenital heart disease	1.77 (1.38-2.28)	<.001	0.69 (0.61-0.78)	<.001
Retransplant	1.85 (1.49-2.29)	<.001	0.70 (0.62-0.80)	<.001
Insurance type				
Private	1 [Reference]	NA	1 [Reference]	NA
Medicare	1.12 (1.01-1.25)	.02	0.98 (0.94-1.03)	.55
Medicaid	1.03 (0.88-1.21)	.63	0.99 (0.93-1.05)	.78
No. of transplant centers per state	0.98 (0.97-0.99)	.01	0.98 (0.96-0.99)	.03
Population density per km^2^	0.99 (0.99-1.00)	.35	0.99 (0.99-0.99)^a^	<.001

Abbreviations: BMI, body mass index (calculated as weight in kilograms divided by height in meters squared); ECMO, extracorporeal membrane oxygenation; HR, hazard ratio; IABP, intra-aortic balloon pump; IV, intravenous; NA, not applicable.

^a^ The hazard ratio for the association between 4 variables (female, BMI, IV inotropic medication, and population) and transplant varied with time. The coefficients of variation were 0.99, 1.0002, 0.99, and 1.00001, respectively, for each unit (ie, day) increase in time (*P* for interaction <.001).

^b^ The hazard ratio for the association between durable MCS and waitlist mortality varied with time, with coefficients of variation of 1.003 (*P* for interaction <.001) for each unit (ie, day) increase in time.

Among patients who underwent heart transplantation, the presence of ECMO at the time of waitlist entry was also the most significant negative factor associated with 1-year graft survival (OR, 0.35; 95% CI, 0.22-0.57) of all recipient characteristics in the SRTR model. Of the donor characteristics evaluated in the model, receipt of heparin before recovery was the most significant positive factor associated with 1-year graft survival (OR, 2.00; 95% CI, 1.39-2.98), while donor to recipient height ratio was the most significant negative factor (OR, 0.21; 95% CI, 0.06-0.71) (Table 3). All results were consistent with the sensitivity analysis using a complete-case approach.

**Table 3.** Multivariable Factors Associated With 1-Year Graft Survival

Characteristic	OR (95% CI)	*P* value
**Recipients**		
Age, y	0.99 (0.98-0.99)	.003
Race/ethnicity		
White	1 [Reference]	NA
Black	1.00 (0.84-1.19)	.96
Hispanic	0.76 (0.60-0.97)	.02
Asian	0.91 (0.62-1.34)	.65
Diabetes	0.86 (0.75-1.00)	.05
BMI	0.90 (0.86-0.95)	<.001
History of cigarette use	0.92 (0.80-1.05)	.23
HCV	0.86 (0.55-1.34)	.52
CMV antibody	1.01 (0.88-1.15)	.91
Previous cancer	0.98 (0.77-1.24)	.87
Previous transplant	0.49 (0.16-1.47)	.20
Total bilirubin, mg/dL	0.89 (0.86-0.93)	<.001
Creatinine, mg/dL	0.94 (0.88-0.99)	.04
Dialysis since waitlist entry	0.57 (0.43-0.75)	<.001
ECMO at transplant	0.35 (0.22-0.57)	<.001
Primary cardiac diagnosis at transplant		
Nonischemic cardiomyopathy	1 [Reference]	NA
Ischemic cardiomyopathy	1.08 (0.77-1.51)	.65
Congenital heart disease	0.48 (0.33-0.71)	<.001
Retransplant	1.18 (0.38-3.64)	.77
Transfusion since waitlist entry	0.72 (0.62-0.82)	<.001
PASP, mm Hg	0.99 (0.98-1.00)	.16
PCW, mm Hg	1.01 (0.99-1.02)	.22
Insurance status		
Primary	1 [Reference]	NA
Medicare	0.92 (0.79-1.07)	.31
Medicaid	1.01 (0.81-1.27)	.86
**Donors**		
Age, y	0.99 (0.98-0.99)	.02
Female sex	0.86 (0.73-1.01)	.06
Blood type group		
A	1 [Reference]	NA
B	0.99 (0.78-1.26)	.98
AB	2.01 (0.93-4.36)	.07
O	0.94 (0.81-1.09)	.44
BMI	1.06 (1.01-1.11)	.009
BUN, mg/dL	1.00 (0.99-1.00)	.27
Cause of death		
Anoxia	1 [Reference]	NA
Cerebrovascular	0.91 (0.75-1.12)	.41
Head trauma	0.96 (0.81-1.14)	.67
CNS tumor	0.65 (0.29-1.50)	.31
High risk for blood-borne disease transmission	1.17 (0.97-1.39)	.08
Insulin-dependent diabetes	0.97 (0.84-1.12)	.72
Ischemic time	0.86 (0.81-0.91)	<.001
Prerecovery heparin use	2.00 (1.39-2.98)	<.001
Donor to recipient height ratio	5.27 (0.34-0.81)	.23
Donor to recipient weight ratio	0.21 (0.06-0.71)	.01

Abbreviations: BMI, body mass index (calculated as weight in kilograms divided by height in meters squared); BUN, blood urea nitrogen; CMV, cytomegalovirus; CNS, central nervous system; ECMO, extracorporeal membrane oxygenation; HCV, hepatitis C virus; NA, not applicable; OR, odds ratio; PASP, pulmonary artery systolic pressure; PCW, pulmonary capillary wedge pressure.

SI conversion factor: To convert BUN to millimoles per liter, multiply by 0.357.

## Discussion

In this analysis of data from the national heart transplantation database in the US, we found significant state-level variation among patients listed at status 1A for the 3 main transplant measures: waitlist mortality, transplant rate, and 1-year graft survival. This variation persisted despite adjustment for donor and recipient characteristics, highlighting the importance of heterogeneity in state-level practices or access to care as factors associated with outcomes.

The exact mechanism underlying state-level variation in waitlist and transplant outcomes is not clear, but it is likely complex and multifactorial. Previous studies have reported substantial geographic variability in donor heart acceptance,^10,11,12^ health care practice or access to care,^13^ and unfair competition for donors with transplantable organs.^14^ In addition, transplant centers are currently being penalized through loss of funding if their reported posttransplant outcomes are below a set standard.^15^ It has also been suggested that the good intentions of this policy to optimize posttransplant outcomes have produced unintended consequences for patients on the waitlist. However, whether these factors could explain state-level variation is not entirely known and requires further research.

Geographic variation in organ matching has been cited as one of the factors associated with changes made to the US heart allocation system in October 2018. Among the main goals of the new 6-tier allocation system were to develop criteria based on illness severity rather than therapeutic interventions and to ensure fair prioritization of recipients with the most serious conditions. Our findings indicating that the presence of ECMO is the most significant factor associated with waitlist mortality and that durable MCS is a factor associated with favorable waitlist outcomes support the prioritization of patients with ECMO into the top tier and the movement of patients with durable MCS into a lower tier in the new allocation system. However, it has also been suggested that the implementation of the new allocation system may have unintended consequences, such as the increased use of temporary MCS and the decreased use of durable MCS.^16,17,18,19^ Hence, prioritization by illness acuity alone may not be the only solution to equity and fairness of organ allocation. In our analysis, state-level variation persisted despite adjustment for multiple patient-level factors of acuity. This finding highlights the importance of other unmeasured potential state-level variables as factors associated with the geographic discrepancy in outcomes. For example, we found that patients living in states with a high number of transplant centers are less likely to die while on the waitlist. Therefore, additional research of factors associated with geographic variation is warranted to further improve fairness in allocation.

Given that patients are generally not able to shop for transplant centers or easily change their state of residence, it is important that the medical community and policy makers reduce the existing interstate gap in transplant outcomes to foster equity and fairness in the allocation system. To our knowledge, the present analysis provides the first insight into the patterns and extent of state-level variation in outcomes, and these data could serve as a reference standard to evaluate the geographic impact, if any, of the new allocation system or any other policy intervention aimed at minimizing disparity in outcomes. Although the new heart allocation policy changed in 2018, many of the geographic and transplant center–associated disparities may persist. The solution to variation in outcomes is likely complex and may not be without unintended consequences. The focus of future research may be best directed toward identifying the factors associated with this variation in outcomes and formulating policies aimed at reducing the interstate gap in outcomes.

### Limitations

This study has several limitations. First, the study is a retrospective analysis of the UNOS database, and our analysis was confined to available variables in the data set. However, we sought additional sources, such as the SRTR registry and the US Census Bureau website, to incorporate additional state-specific data. Although we adjusted for a comprehensive list of potential covariates in our analysis, there is the possibility that residual confounding occurred because of unmeasured variables. Second, the exact mechanism underlying the interstate gap in outcomes could not be ascertained in this analysis. Therefore, further research will be helpful to identify and address these mechanistic factors. Third, the donor heart allocation system was revised in 2018 from a 3-tiered system (ie, status 1A, 1B, and 2A) to a 6-tiered system (ie, status 1-6), such that patients who were included in the status 1A tier before 2018 are now stratified into status 1 through status 3 tiers. However, this policy does not directly address state-specific variations in practice that may underlie interstate variation in outcomes.

## Conclusions

In this study, significant state-level variation was found in waitlist mortality, transplant rates, and 1-year graft survival among patients listed for heart transplantation in the US between 2011 and 2016. Identifying and addressing the factors associated with these geographic variations in outcomes is important to ensure a fair allocation system.

## Notes

Supplement. **eTable 1.** Variables in the SRTR Risk Adjustment Model for Posttransplant Outcomes **eTable 2.** Outcomes by State (Arranged From Lowest to Highest) **eTable 3.** Relative Estimates for Each End Point by Quartile of States **eTable 4.** Two-Letter Abbreviations of States, Stratified by OPTN Region
