# Supplementary material for: State-Level Variation in Waitlist Mortality and Transplant Outcomes Among Patients Listed for Heart Transplantation in the US From 2011 to 2016
Source: JAMA Netw Open. 2020 Dec 9;3(12):e2028459. doi: 10.1001/jamanetworkopen.2020.28459 (PMC7726636; doi:10.1001/jamanetworkopen.2020.28459)
Supplement: Supplement. — eTable 1. Variables in the SRTR Risk Adjustment Model for Posttransplant Outcomes eTable 2. Outcomes by State (Arranged From Lowest to Highest) eTable 3. Relative Estimates for Each End Point by Quartile of States eTable 4. Two-Letter Abbreviations of States, Stratified by OPTN Region [file jamanetwopen-e2028459-s001.pdf]

Supplementary Online Content

Akintoye E, Shin D, Alvarez P, Briasoulis A. State-level variation in waitlist mortality and transplant outcomes among patients listed for heart transplantation in the US from 2011 to 2016. *JAMA Netw Open*. 2020;3(12):e2028459. doi:10.1001/jamanetworkopen.2020.28459

eTable 1. Variables in the SRTR Risk Adjustment Model for Posttransplant Outcomes

eTable 2. Outcomes by State (Arranged From Lowest to Highest)

eTable 3. Relative Estimates for Each End Point by Quartile of States

eTable 4. Two-Letter Abbreviations of States, Stratified by OPTN Region

This supplementary material has been provided by the authors to give readers additional information about their work.

© 2020 Akintoye E et al. *JAMA Network Open*.

eTable 1. Variables in the SRTR Risk Adjustment Model for Posttransplant Outcomes

**Recipient characteristics**
Age, years
Ethnicity
Diabetes
BMI, kg/m^2
History of cigarette use
HCV
CMV antibody
Previous malignancy
Previous transplant
Total bilirubin, mg/dL
Creatinine, mg/dL
Dialysis since listing
ECMO at transplant
Primary cardiac diagnosis at transplant
Non-ischemic cardiomyopathy
Ischemic cardiomyopathy
Congenital heart disease
Re-transplant
Transfusion since listing
PASP, mmHg
PCW, mmHg
Insurance status
Primary
Medicare
Medicaid
**Donor characteristics**
Age, years

© 2020 Akintoye E et al. *JAMA Network Open*.

Female
Donor ABO status
BMI, kg/m2
BUN, mg/dL
Cause of death
Anoxia
Cerebrovascular
Head trauma
CNS tumor
High risk for blood-borne disease transmission
Insulin-dependent diabetes
INR
Ischemic time
Pre-recovery heparin use
Donor-recipient height ratio
Donor-recipient weight ratio

© 2020 Akintoye E et al. *JAMA Network Open*.

eTable 2. Outcomes by State (Arranged From Lowest to Highest)

Order	Person-days on waitlist at 1A		No of deaths on waitlist per 1000 person-days		No. of transplant per 1000 person-days		Risk-adjusted 1-year graft survival rate, %	
1	AK	514	NE	1.0	VT	5.6	NV	87
2	HI	624	WY	1.0	SD	5.8	AK	87
3	MT	758	WV	1.2	RI	6.2	ID	87
4	WY	953	RI	1.3	MA	6.6	WY	87
5	ID	1214	NH	1.3	NH	6.7	LA	87
6	NV	1662	ND	1.4	DC	7.4	VA	88
7	NM	1687	ME	1.4	NY	7.4	CO	88
8	DE	1713	VT	1.5	ME	7.4	ND	88
9	KS	2147	MA	1.6	CT	7.6	NJ	88
10	UT	2815	HI	1.6	ND	7.9	IA	88
11	VT	3376	NY	1.7	VA	8.2	TX	88
12	ND	3653	CT	1.7	GA	8.8	FL	88
13	CO	3844	UT	1.8	MN	9.2	NM	88
14	NE	3951	VA	1.8	IA	10.3	CA	88
15	SD	3986	IL	2.0	MO	11.4	NE	88
16	WV	4170	DC	2.1	KY	11.9	OR	88
17	SC	4340	IA	2.2	IL	12.1	KY	88
18	OK	4684	SD	2.3	MD	12.1	NY	88
19	DC	4727	MO	2.3	AR	13.8	WA	88
20	ME	4835	MN	2.5	AL	14.3	WV	88
21	MS	5309	AZ	2.7	WY	14.7	OH	88
22	AL	5331	WI	2.7	MS	14.7	AZ	89
23	RI	5489	WA	2.7	FL	15.1	MN	89
24	OR	5997	CO	2.9	WI	15.2	SD	89
25	AR	6600	PA	2.9	WA	15.6	SC	89

© 2020 Akintoye E et al. *JAMA Network Open*.

26	AZ	7489	OH	2.9	SC	15.7	PA	89
27	NH	8183	DE	2.9	WV	15.8	CT	89
28	LA	9178	NM	3.0	TX	16.3	VT	89
29	IA	10103	OR	3.0	OR	17.0	AL	89
30	TN	12100	GA	3.0	PA	17.4	UT	89
31	WA	12591	FL	3.2	NC	17.6	MS	89
32	KY	13363	MI	3.3	LA	17.7	MA	89
33	IN	13550	TX	3.3	OH	17.7	ME	89
34	MO	13740	IN	3.3	MI	17.9	WI	89
35	NJ	14386	NC	3.6	TN	18.4	AR	89
36	WI	17407	CA	3.6	OK	19.2	IL	89
37	MI	19033	AR	3.6	DE	20.4	MT	89
38	OH	19078	MD	3.7	MT	21.1	OK	89
39	MD	19105	KS	3.7	NM	21.3	NC	89
40	MN	21000	AL	3.8	CO	21.9	GA	89
41	NC	21650	NJ	3.8	AZ	23.1	DE	89
42	CT	24283	MS	3.8	IN	23.5	IN	89
43	PA	27581	KY	4.0	NE	23.8	NH	89
44	GA	30562	ID	4.1	UT	26.3	TN	89
45	MA	37025	OK	4.5	CA	26.9	MD	89
46	FL	40307	NV	4.8	AK	27.2	MO	90
47	IL	45431	MT	5.3	ID	28.0	RI	90
48	CA	47286	TN	5.3	HI	28.8	DC	90
49	VA	47348	LA	5.7	NJ	29.1	MI	90
50	TX	67181	SC	5.8	NV	29.5	KS	90
51	NY	113147	AK	7.8	KS	34.5	HI	92

© 2020 Akintoye E et al. *JAMA Network Open*.

eTable 3. Relative Estimates for Each End Point by Quartile of States

	HR/OR*	95% CI	*P* value
Quartile of waitlist mortality			
1st quartile	Reference		
2nd quartile	1.04	0.94-1.17	0.49
3rd quartile	1.01	0.89 -1.14	0.96
4th quartile	1.53	1.27-1.86	<0.001
Quartile of transplant			
1st quartile	Reference		
2nd quartile	1.00	0.93-1.07	0.89
3rd quartile	1.11	1.01-1.24	0.03
4th quartile	1.57	1.31-1.87	<0.001
Quartile of 1-year graft survival			
1st quartile	Reference		
2nd quartile	1.28	1.09-1.50	0.002
3rd quartile	1.60	1.33-1.92	<0.001
4th quartile	2.07	1.64-2.62	<0.001

Abbreviations. HR, hazard ratio; OR, odds ratio; CI, confidence interval

*Estimates are hazard ratio for waitlist mortality and transplant; and odds ratio for 1-year graft survival

© 2020 Akintoye E et al. *JAMA Network Open*.

eTable 4. Two-Letter Abbreviations of States, Stratified by OPTN Region

OPTN region	State	Abbreviation	OPTN region	State	Abbreviation
1	Connecticut	CT	7	Illinois	IL
1	Massachusetts	MA	7	Minnesota	MN
1	Maine	ME	7	North Dakota	ND
1	New Hampshire	NH	7	South Dakota	SD
1	Rhode Island	RI	7	Wisconsin	WI
2	District of Columbia	DC	8	Colorado	CO
2	Delaware	DE	8	Iowa	IA
2	Maryland	MD	8	Kansas	KS
2	New Jersey	NJ	8	Missouri	MO
2	Pennsylvania	PA	8	Nebraska	NE
2	West Virginia	WV	8	Wyoming	WY
3	Alabama	AL	9	New York	NY
3	Arkansas	AR	9	Vermont	VT
3	Florida	FL	10	Indiana	IN
3	Georgia	GA	10	Michigan	MI
3	Louisiana	LA	10	Ohio	OH
3	Mississippi	MS	11	Kentucky	KY
4	Oklahoma	OK	11	North Carolina	NC
4	Texas	TX	11	South Carolina	SC
5	Arizona	AZ	11	Tennessee	TN
5	California	CA	11	Virginia	VA
5	New Mexico	NM			
5	Nevada	NV			

© 2020 Akintoye E et al. *JAMA Network Open*.

5	Utah	UT			
6	Alaska	AK			
6	Hawaii	HI			
6	Idaho	ID			
6	Montana	MT			
6	Oregon	OR			
6	Washington	WA			

© 2020 Akintoye E et al. *JAMA Network Open*.
